# Supplementary figures and images for: The electrogenicity of the Na+/K+-ATPase poses challenges for computation in highly active spiking cells
Source: eLife. 2025 Dec 3;14:RP103781. doi: 10.7554/eLife.103781 (PMC12674616; doi:10.7554/eLife.103781)

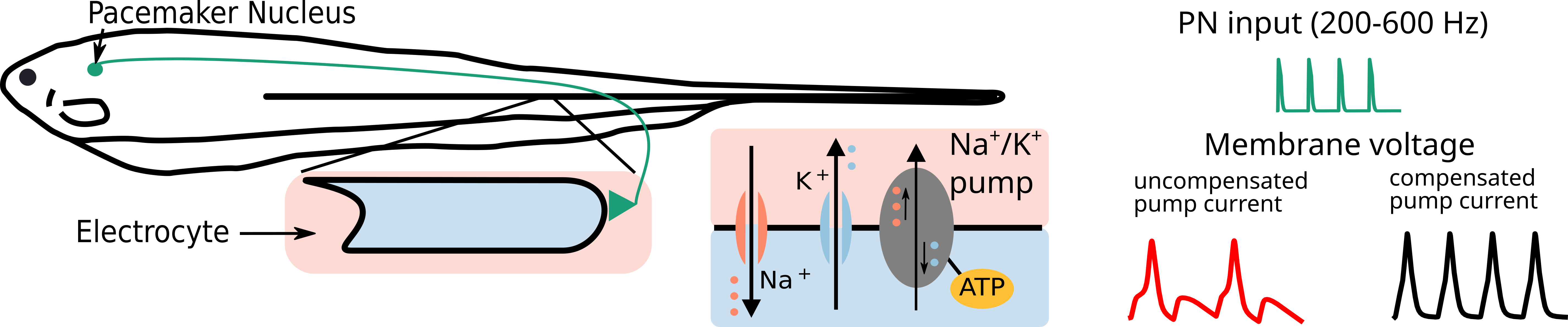

Supplement: Source code 1. [file elife-103781-code1.zip › electrocyte_nakatpase-main/fig/img/electrocyte_model.png]

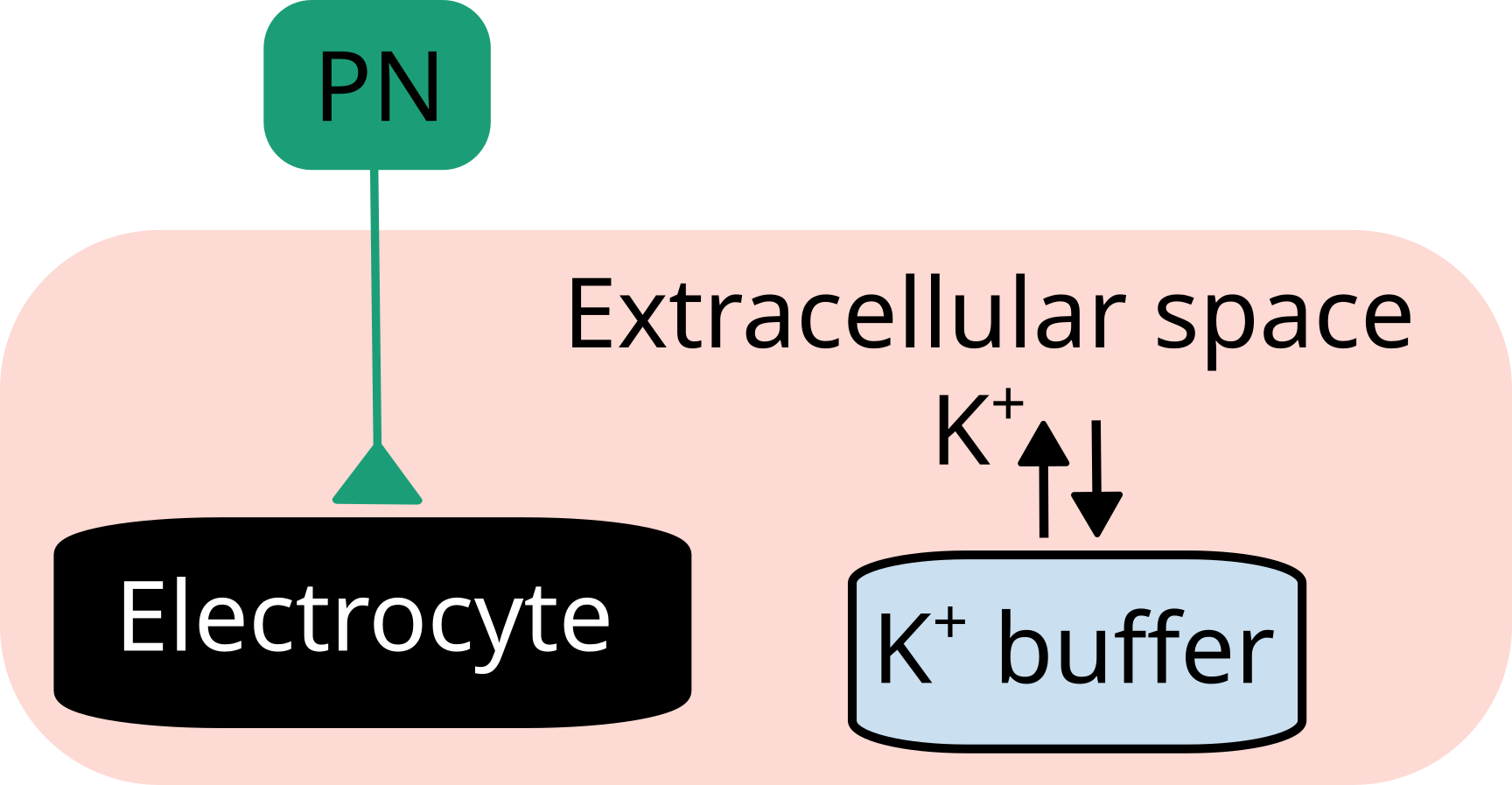

Supplement: Source code 1. [file elife-103781-code1.zip › electrocyte_nakatpase-main/fig/img/k_buffer.png]

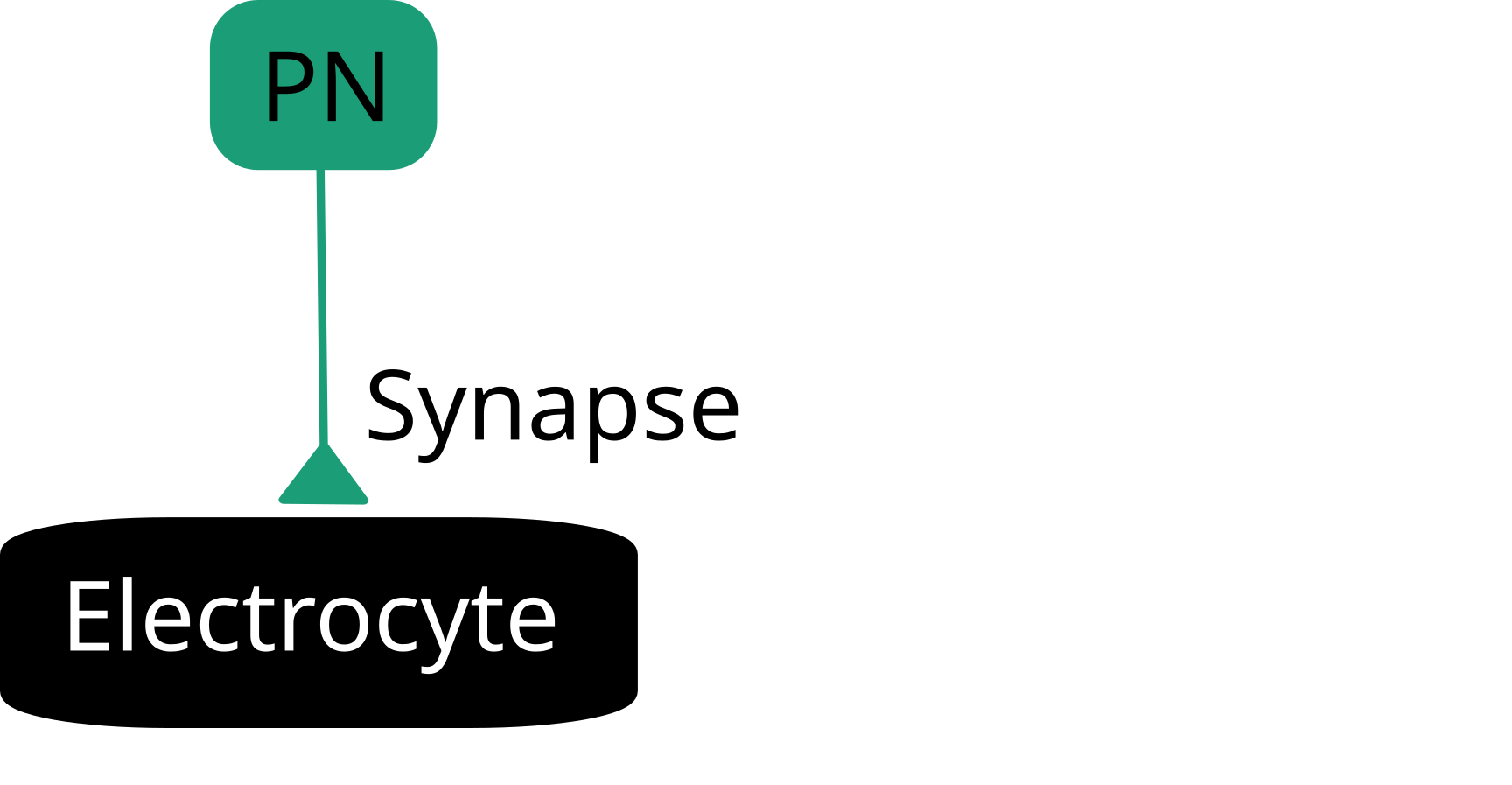

Supplement: Source code 1. [file elife-103781-code1.zip › electrocyte_nakatpase-main/fig/img/synapse.png]
